# Choroidal and Retinal Thickness of Highly Myopic Eyes with Early Stage of Myopic Chorioretinopathy: Tessellation

**DOI:** 10.1155/2018/2181602

**Published:** 2018-02-11

**Authors:** Yanping Zhou, Minlu Song, Minwen Zhou, Yiming Liu, Fenghua Wang, Xiaodong Sun

**Affiliations:** ^1^Department of Ophthalmology, Shanghai General Hospital (Shanghai First People's Hospital), Shanghai Jiao Tong University School of Medicine, Shanghai, China; ^2^Shanghai Engineering Center for Visual Science and Photomedicine, Shanghai, China; ^3^Shanghai Key Laboratory of Fundus Diseases, Shanghai, China

## Abstract

**Purpose:**

To investigate the choroidal thickness (CT) and retinal thickness (RT) in highly myopic tessellated eyes.

**Methods:**

In this study, 115 highly myopic eyes were recruited and divided as tessellated fundus (*n* = 93) and normal fundus (*n* = 22). RT and CT were quantified using optical coherence tomography with enhanced depth imaging (EDI-OCT). Correlation between subfoveal CT (SFCT) and tessellation was analyzed using logistic regression models.

**Results:**

Tessellated fundus eyes had thinner CT than did normal fundus eyes, while RT was not statistically different across groups. The tessellated eyes had a thinner choroid than did the control eyes at all measured macular locations (all *P* < 0.05). After adjustment for AL, age, and gender, the SFCT was significantly associated with tessellation. The odds ratio (OR) and 95% confidence interval (CI) was 0.975 (0.960–0.990, *P* = 0.001, binary logistics regression) and 0.991 (0.984–0.999, *P* = 0.022, Cox regression). The area under the curve (AUC) of SFCT was the greatest for detecting tessellation (AUC = 0.824, *P* < 0.001). For sensitivity and specificity analyses, SFCT had the highest diagnostic value (sensitivity = 81.8%, specificity = 74.2%).

**Conclusions:**

Highly myopic eyes with tessellation have thinner CT than do normal highly myopic eyes. CT may serve as an early pathologic predictor of high myopia.

## 1. Introduction

High myopia is a common global public health problem. Its prevalence varies from 2.4% in the USA [1], 4.2% in Taiwan [2], and 8.2% in Japan [3]. Worldwide, the prevalence of adult myopic chorioretinopathy ranges from 1.2 to 3.1% [2, 4–6]. Further, the prevalence of myopia is particularly as high as 95.5% in a population of Chinese university students [7]. Given such high prevalence of myopia and myopic chorioretinopathy, the earlier-stage characteristics of pathologic myopia is warranted.

Recently, Ohno-Matsui et al. [8] have summarized the pathologic myopic macular chorioretinopathy and graded them from grade C_0_ to C_5_ to describe the increasing severity. Tessellated fundus is classified into category 1 (C_1_) and is the earliest pathologic chorioretinal change of pathologic myopic eyes [8], similar to the Avila classification [9]. One of the most common longitudinal progression patterns observed in a long-term study of myopia (mean follow-up time of 12.7 years) [10] was for tessellated fundus to develop lacquer cracks and diffuse atrophy. Wang et al. [11] reported a choroid thinning in eyes with lacquer crack. The choroid, given its special position between sclera and retina and its role of offering nutrient and oxygen for outer retina [12], is potentially important in the process of myopic chorioretinopathy. However, it is unknown how choroidal thinning is associated with the initial stages (C_1_: tessellation) of pathologic myopia.

To characterize the choroid and to evaluate early tessellation changes in myopic chorioretinopathy, we quantitatively assess the retinal thickness and choroidal thickness using EDI-OCT in highly myopic eyes with normal visual acuity.

## 2. Materials and Methods

### 2.1. Design and Patients

A cross-sectional study was performed to observe choroid and retina features of highly myopic subjects diagnosed as tessellation fundus with normal visual acuity using EDI-OCT. This study was approved by the Ethical Review Committee of the Shanghai General Hospital affiliated with Shanghai Jiao Tong University and was in accordance with the Declaration of Helsinki. All subjects signed an informed consent form after an explanation of the purpose and procedures of the OCT examination.

Major study ocular eligibility criteria include the following: (1) phakic eyes with best-corrected visual acuity (BCVA) ≥ 20/20 and refractive error worse than −6 diopter (D); (2) adults (older than 18 years); and (3) no myopic retinal degenerative lesion (C_0_) or only tessellated fundus (C_1_) related to myopia [8]. Tessellated fundus is defined as the condition in which the choroidal vessels can be seen through the retina owing to reduced pigmentation or hypoplasia of the retinal pigment epithelium (RPE) [13].

Patients with history of hypertension or diabetes, confounding ocular disease, additional eye operations, including vitreoretinal or cataract surgery or refractive surgeries, and other myopic complications (including lacquer cracks, posterior staphyloma, choroidal atrophy, choroidal neovascularization, subretinal hemorrhage, macular traction, macular hole, retinal detachment, and retinoschisis) were excluded.

### 2.2. Eye Examinations

The following were performed before the OCT scanning: (1) evaluation of refractive error without pupil dilation by optometry (AR-310A, NIDEK, Japan); (2) axial length measurement by IOLMaster (Carl Zeiss Meditec AG, Germany); (3) intraocular pressure measurement by noncontact tonometer (TX-20, Canon, Japan); (4) color fundus photography (retinal camera CR-DGi, Canon, Japan); (5) ultrasonography of the eye ball to exclude posterior staphyloma (B-scan CineScan, Quantel Medical, France); and (6) careful examination of the peripheral retina by a senior retinal specialist (Fenghua Wang) using an indirect ophthalmoscope to exclude peripheral degeneration, retinal tear, or retinal detachment.

### 2.3. EDI-OCT Scanning and Measurements

**Figure 1:**
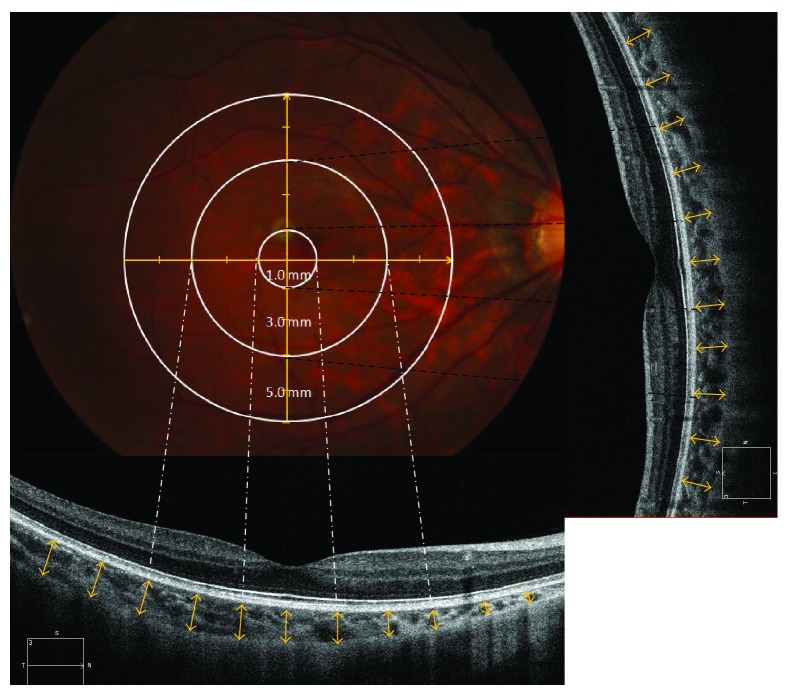
Examples of photographs of the posterior pole and spectral domain optical coherence tomography (SD-OCT) in highly myopic eyes. Fundus photograph (top left) and EDI-OCTs in the horizontal direction (bottom) and in the vertical direction (right). This is a tessellated fundus of a 21-year-old female with refractive error equal to −8.75 diopter and 27.24 mm elongation of the eye ball. Both horizontal (bottom) and vertical scans (right) across the fovea of this tessellated myopic eye. The yellow cross in the fundus photograph shows the scan protocol of high-resolution scan with enhanced depth imaging. The white and black dotted lines refer to the ETDRS auxiliary lines. The white concentric circles have diameters of 1.0 mm (inner), 3.00 mm (middle), and 5.0 mm (outer). Although the scan length was set to 6 mm, the actual measurements were stopped in loci of 5.0 mm because of a little missing image. The yellow double-headed arrows in OCT scans demonstrate the measured loci of the 500 *μ*m intervals.

Choroidal thickness was measured using the built-in EDI Mode of the Cirrus High-Definition SD-OCT (model 4000, software version 5.2; Carl Zeiss Meditec, Dublin, CA) with the instrument close enough to the eye to obtain an inverted image. A 5-line (6 mm) high-resolution raster scan was taken of each eye in two directions (horizontal and vertical) by an experienced operator. Also, a macular cube scanning of 512∗128 was operated for an exclusion of other maculopathies in the posterior pole. Measurements from the scans were taken as follows: (1) choroidal thickness (CT) was measured from the outer portion of the hyperreflective line corresponding to the RPE to the inner surface of the sclera (Figure 1) [11]; (2) central retinal thickness was measured from the inner limiting membrane to the outer border of the RPE; (3) CT measurements were made at an interval of 0.5 mm from central fovea to superior, inferior, temporal, and nasal sides (S, I, T, and N); (4) central subfield thickness was the mean thickness of the central 1 mm subfield according to the Early Treatment of Diabetic Retinopathy Study Grid, which was automatically measured by the built-in software with manual correction of segmentation or foveal location permitted; (5) macular choroidal thickness is referred to as the average of choroidal thickness in different locations. Each measurement was independently performed by two observers (Yanping Zhou and Yiming Liu) for repeated-measures analysis of variance.

### 2.4. Diagnosis of Tessellation

Two of our authors (Minlu Song and Minwen Zhou), masked from patients' basic information and OCT scanning, graded the color fundus photographs independently into normal fundus (C_0_) and tessellated fundus (C_1_) based on the classification reported by Ohno-Matsui et al. [8]. Divergences were finally approved by another senior retinal specialist (Xiaodong Sun).

### 2.5. Statistical Analysis

All of the data were analyzed by a statistical software program (SPSS 18.0; SPSS, Inc., Chicago, IL), and results presented are the mean ± standard deviation (SD). The Kolmogorov-Smirnov test was used to decide normal distributions. Independent *t*-test was performed to test the between-group comparisons. One-way ANOVA was applied to compare mean thickness in different locations. Then, multiple linear regression analysis using stepwise selection method was used to see the association factors with SFCT. We also used logistic regression models to determine the risk factors of tessellation. Further, age- and gender-matched subgroup analysis was performed to determine the risk factors of tessellation using Cox regression. What is more, receiver operating characteristic (ROC) curves were generated, and the area under the curve (AUC) was applied to assess the property of biometric parameters in perceiving highly myopic tessellation. *P* < 0.05 was considered statistically significant in all tests.

## 3. Results

### 3.1. Basic Information

There were 67 Chinese subjects from Shanghai included for our study, both eyes of the patients were examined, and we excluded 19 eyes with the following conditions: 6 eyes cannot gain enough signal strength of 5 lines in the HD-Scan, 3 eyes had lacquer cracks, and 10 eyes had BCVA less than 20/20. 93 eyes were graded as C_1_ (group C_1_: tessellated fundus), and 22 eyes were graded as C_0_ (group C_0_: normal fundus). Therefore, data from 93 eyes with tessellation and 22 eyes nontessellation with high-quality OCT images were available and were included in the statistical analysis.  Table 1 presents the basic features of the two groups. There were significant differences between the two groups for axial length and spherical equivalent refraction (SER) (*P* < 0.05). As expected, the highly myopic eyes with tessellation had longer axial length and worse refractive errors. There was no significant difference in mean age and intraocular pressure (IOP) between the two groups.

### 3.2. Choroidal Thickness in Different Locations

**Figure 2:**
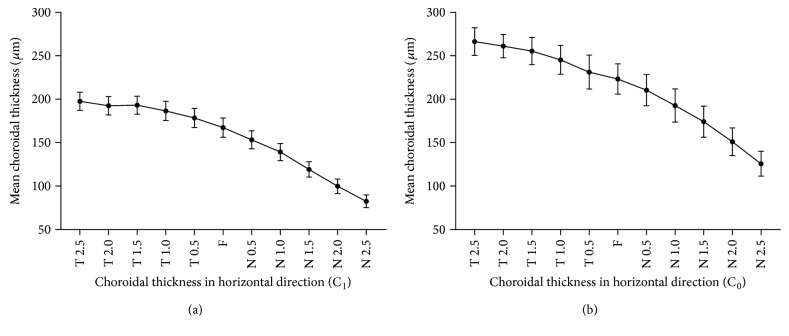
Mean choroidal thickness of group C_1_ and group C_0_ in the horizontal direction. Group C_1_ is referred to as the highly myopic eyes with only tessellated fundus in the posterior pole, while group C_0_ is referred to as the normal highly myopic eyes. Both groups have a tendency for choroid thinning from the temporal side to the nasal side. Eyes with tessellation fundus have much thinner choroid in each locus in the horizontal direction.

**Figure 3:**
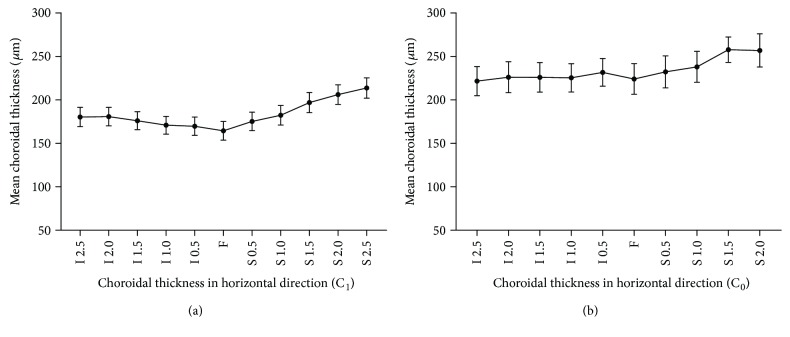
Mean choroidal thickness of group C_1_ and group C_0_ in the vertical direction. Choroidal thickness of normal highly myopic eyes in the vertical direction is thicker than that of highly myopic eyes with only tessellation fundus. Choroid becomes thicker from central to the surrounding of group C_1_. And the central subfoveal choroidal thickness of group C_1_ is the thinnest in the vertical direction. However, choroidal thickness in group C_0_ was much lesser than that in group C_1_. The central subfoveal choroidal thickness thins the most between the two groups.

The mean subfoveal choroidal thickness (SFCT) was 165.9 *μ*m (SD, ±52.4) in the tessellation eyes and 223.6 *μ*m (SD, ±39.3) in the normal fundus eyes (*P* < 0.001). Relatedly, the macular choroidal thickness (MCT) was 169.4 *μ*m (SD, ±45.1) in the tessellation eyes and 224.3 *μ*m (SD, ±29.1) in the normal fundus eyes (*P* < 0.001). Choroidal thickness (CT) decreased horizontally from temporal to nasal positions as showed in Figure 2. And the CT in the vertical direction is shown in Figure 3.  Table 2 demonstrates the mean CT of these two groups at different loci in the horizontal direction. And Table 3 demonstrates the corresponding data in the vertical direction. The choroidal thickness was significantly thinner in the tessellated fundus than in the normal fundus of highly myopic eyes in all locations (all *P* < 0.05).

### 3.3. Retinal Thickness

The retinal thickness was also compared between the two groups. The foveal thickness was 191.2 *μ*m (SD, ±15.0) in the tessellation eyes and 192.9 *μ*m (SD, ±14.7) in the normal fundus eyes (*P* = 0.626). Also, their macular retinal thickness was not statistically significant, with *P* value = 0.360 (*P* > 0.05). We conclude that the retina thickness was similar between the two groups.

### 3.4. Stepwise Multiple Linear Regression Analysis

As choroidal thickness was significantly different between the two groups as mentioned before, we speculated that it may be a risk factor of tessellation. Therefore, we performed stepwise analysis to find out factors associated with the SFCT. Age, gender, axial length, and the tessellation diagnosis were included in this model.  Table 4 exhibits the results of multiple regression analysis. The tessellation classification was associated with SFCT (*P* < 0.001) after adjusting for the axial length, age, and gender by multiple linear regression analysis. We now calculated that between tessellation and nontessellation eyes; the latter had a thicker SFCT averaged as 54.61 *μ*m than had tessellation eyes (*P* < 0.001). Likewise, this model was also used for other locations.  Table 5 shows the detailed data in various locations. The differences in all locations remained statistically significant (*P* < 0.05).

### 3.5. Risk Factors Causing Tessellation

Further, we made binary logistic regression models to determine the risk factors of highly myopic tessellation. After screening for age, gender, axial length, refractive error, SFCT, and foveal retinal thickness for tessellation, there was a statistically significant association between increased axial length and decreased SFCT as listed in Table 6. After the multivariate logistic regression, the multivariate-adjusted ORs of axial length and SFCT for highly myopic tessellation still remained statistically significant.

### 3.6. Age- and Gender-Matched Subgroup Analysis

In the present study, we performed 2 : 1 age- and gender-matched case-control subgroup analysis to evaluate the potential risk factors associated with myopic tessellation. Age was matched as ±3 years.  Table 7 demonstrates the basic information of each subgroup. Additionally, conditional logistic regression model (Cox regression) was performed. After adjusting for the age, gender, axial length, refractive error, SFCT, and foveal retinal thickness, there was a significant association between increased axial length (OR 2.001, 95% CI 1.042–3.843, *P* = 0.037) and decreased SFCT (OR 0.991, 95% CI 0.984–0.999, *P* = 0.022).

### 3.7. AUC Analysis of Ocular Parameters for Detecting Tessellation in Highly Myopic Eyes

The AUC, sensitivity, and specificity of ocular parameter were analyzed to diagnose highly myopic tessellation.  Table 8 summarizes these results. The largest AUC was referred to SFCT (AUC = 0.824), and the AUC of axial length (AUC = 0.820) was rank only second to SFCT, followed by refractive error (AUC = 0.639). However, it was noted that foveal retinal thickness was not statistically significant for diagnosing highly myopic tessellation (AUC = 0.528, *P* = 0.688). For the sensitivity and specificity analyses, SFCT had the highest diagnostic value (sensitivity = 81.8%, specificity = 74.2%).

## 4. Discussion

Previous data have shown the ocular predictors for high myopia including a worsening of refractive error, an extension of axial length, and thinning of retina and choroid [14, 15]. Besides, female gender, older age, ethnicity of developing countries, and less outdoor exercise lifestyle are the demographic risk factors [16]. A previous study also showed that the thinning choroid was the main ocular predictor for lacquer cracks [11]. The present study examines the choroidal features and retinal thickness of high myopia in early stage: tessellation. And it was suspected that the thinning of choroid is the risk factor of great concern for tessellation.

As present in Table 1, compared with the normal highly myopic eyes (C_0_ group), pathologic myopic eyes with a tessellated fundus (C_1_ group) had significantly longer axial length, more myopia, and thinner choroid but no significant difference in retinal thickness. Previous EDI-OCT-based studies have associated choroidal thickness changes in pathologic myopic eyes with lacquer cracks [11] and choroidal neovascularization [17, 18]. Also, Wang et al. [19] compared choroidal thickness in pathologic myopic eyes in early classification of dry-type myopic maculopathy which were with a tessellated fundus and with diffuse chorioretinal atrophy. Better BCVA, less myopia, shorter axial length, and less staphyloma were found in tessellation eyes than in diffuse chorioretinal atrophic eyes. Relatedly, our study specifically focused on these young subjects who had only tessellation fundus in the posterior pole (which is graded as C_1_, the earliest fundus changes of pathologic myopia), allowing us to quantify choroidal thickness changes and to infer potential early pathologic prognostic indicators. Foveal retinal thickness of C_0_ group and C_1_ group had no statistically significant changes. Even foveal retinal thickness remained in the normal range despite a significant decrease in SFCT in C_1_ group compared with C_0_ group [20, 21], suggesting that choroidal thinning may occur before retinal thinning in the early stage of high myopia and that accelerated choroidal thinning may result in a series lesions of maculopathy. Thus, knowing about the role of choroid may gain a better understanding of the start of pathologic myopia.

In addition to central subfoveal choroidal thickness, we also measured horizontal and vertical variations in CTs in the macula. It was observed that the choroid gradually became thinner from the temporal to nasal side, similar to what was observed in several other studies of horizontal variation in CT of highly myopic eyes [18, 19, 22, 23]. Both C_1_ group and C_0_ group had this tendency. Comparing choroidal thickness at each corresponding locations of C_1_ group with C_0_ group, we found that it was significantly thinner in eyes with tessellation changes. However, previous studies of normal eyes indicate that the choroid is thickest at the fovea in the horizontal direction [20, 21]. Ohsugi et al. compared the CT of otherwise normal highly myopic eyes and normal eyes using three-dimensional tomography and found that the choroid in all regions of myopic eyes is thinner than in normal eyes and only the outer nasal choroid was significantly thinner than the central subfoveal choroid [24]. The blood supply for the choroid originates from the posterior ciliary arteries in the macula and then travels to the so-called watershed zones of the choroid periphery, which may be the reason for the reduction in CT towards the outer nasal regions [25]. This may also explain why the pathologic myopia-associated depigmentation known as myopic conus first occurs on the temporal side of the optic disc.

On the vertical direction, we found that the choroid was thinnest at the central subfovea in tessellation eyes while there was no significant difference between central subfoveal choroidal thickness and inferior choroidal thickness in normal highly myopic eyes. However, Ding et al. [26] found that the choroid was the thickest underneath the fovea in Chinese normal eyes. Those variations might change with the process of myopia. Elongation of axial length may explain the phenomenon of greater choroidal thinning in the central fovea versus thinning found in the regions inferior and superior to the central fovea. Axial length is referred as to the distance between cornea and central fovea. Whether choroidal thinning leads to globe elongation or the inverse remains unknown. Visual signal stimulation in fovea may be another risk factor influencing the decrease of central subfoveal choroidal thickness. A brief but infrequent period of hyperopic defocus from negative lenses showed choroidal thinning without axial elongation in experimental chick eyes [27]. Since no experimental research could take place in human eyes without invasion, longitudinal observation of tessellation is needed.

As it is known that C_1_ group eyes had decreased choroidal thickness in comparison with C_0_ group eyes, multiple regression analysis revealed that the SFCT of C_1_ group eyes was 54.61 *μ*m on average thinner than that of C_0_ group eyes. After adjusting of axial length, gender, age, and refractive error by multivariate linear regression analysis, the mean SFCT of C_1_ group eyes still remained statistically significantly thinner than that of the C_0_ group eyes. Furthermore, we also found that the decreased choroidal thickness was independently associated with myopic tessellation after adjusting for AL, age, gender, and RE, which indicated choroid thinning may be relatively the most important factor for tessellation. Until now, no one can figure out the AL elongation and choroid thinning, the two factors which take place earlier and had more powerful influence in the formation of tessellation. However, our study proved that choroid thinning played a more important role in tessellation. The thinning of choroidal thickness does not certainly present the occurrence of tessellation. It is only one stage of tessellation formation.

Even though there were lots of studies on highly myopic choroidal thickness, there were no such quantitative analyses of choroidal thickness being associated with tessellation. In this study, both multivariate logistic regression and age- and gender-matched Cox regression demonstrated that the thinner SFCT and longer axial length had significant association with tessellation. So increased SFCT seemed to be a protective factor for tessellation; in turn, the decreased SFCT may be one risk factor for tessellation. Statistically, these findings were significant; longer axial length and thinner SFCT eyes had greater risks for forming tessellation. These two parameters could be the main parameters for the diagnosis of tessellation.

Last but not least, the area under the curve (AUC) predicted that pathologic myopic tessellation–associated choroid degeneration in the posterior pole of the eye occurred before retina degeneration. We made AUC analysis to assess SFCT, AL, RE, and FRT in detecting highly myopic tessellation. The main AUC of SFCT was the highest, which was 0.824, 95% CI (0,747, 0.901), followed by axial length and then refractive error, which were statistically significant for detecting highly myopic tessellation. However, the AUC of foveal retinal thickness was not statistically significant, which was 0.528, 95% CI (0.390, 0.666). What is more, SFCT and axial length had relatively great sensitivity (81.8% of SFCT) and specificity (77.3% of axial length), which means SFCT, as one of the most important parameter, may play a great role in detecting highly myopic tessellation.

Nevertheless, this study has some limitations. Firstly, there was lack of prospective observations. Secondly, as all of the measurements were performed manually, the determination of the sclera border was somewhat subjective. Thirdly, Tan et al. [28] had reported that choroidal thickness was significantly different diurnally in normal healthy subjects. We did not scan the subjects' choroid at the same time, but all of the OCT scanning was taken near noontime. Lastly, there may be some residual error by not taking into account axial length to correct for ocular magnification in this paper [29]. Also, as only tessellated pathologic myopic eyes are usually less myopic and have shorter axial length than does severe chorioretinal atrophy, the refractive error and axial length are narrowly distributed in our study. Thus, correlation of their choroidal thickness has no significant difference, which differs from other previous studies. Ikuno et al. [30] and Ohno-Matsui et al. [31] have demonstrated that pathologic degeneration starts at the choriocapillaris layer, Bruch's membrane, and RPE layer. Myopia-related stretching of axial length and the ischemia and hypoxias resulting from hypoperfusion may cause tearing of Bruch's membrane and alter the structure of RPE cells [32]. However, it remains unclear how choroidal thinning is associated with the beginning of this chorioretinal degeneration. Further prospective studies are needed to characterize longitudinal changes in the choroid, RPE layer, and outer retina in highly myopic eyes. Such a follow-up study would focus on the role of choroidal changes in the progression of pathologic myopia.

In conclusion, choroid thins in highly myopic tessellated eyes than in normal highly myopic eyes while retina does not, suggesting an important role of the choroid in the early stage of myopic chorioretinopathy. Early quantitative assessment of choroidal thickness and qualitative examination of choroid morphology can be predictive of myopic maculopathy.

## Figures and Tables

**Table 1:** Basic biometrics of tessellation eyes and normal fundus eyes.

	Tessellated fundus eyes	Normal fundus eyes	*P* value
No. of eyes	93	22	—
Age, year	34.3 ± 10.5	37.2 ± 9.9	0.239
IOP at imaging, mmHg	15.81 ± 2.26	16.01 ± 2.59	0.724
Axial length, mm	27.04 ± 0.71	26.12 ± 0.74	<0.001^∗^
Spherical equivalent, diopter	−8.813 ± 1.647	−7.882 ± 1.017	0.025^∗^
Subfoveal choroidal thickness, *μ*m	165.9 ± 52.4	223.6 ± 39.3	<0.001^∗^
Macular choroidal thickness, *μ*m	169.4 ± 45.1	224.3 ± 29.1	<0.001^∗^
Foveal retinal thickness, *μ*m	191.2 ± 15.0	192.9 ± 14.7	0.626
Central subfield thickness, *μ*m	249.2 ± 18.9	245.0 ± 21.0	0.360

Data are demonstrated as mean ± SD. IOP: intraocular pressure. ^∗^*P* value < 0.05.

**Table 2:** Mean choroidal thickness in the horizontal direction.

Location (mm from fovea)	Tessellated fundus eyes	Normal fundus eyes	*P* value
	Mean ± SD, *μ*m	Mean ± SD, *μ*m	
Temporal (2.5)	197.6 ± 50.3	266.3 ± 35.9	<0.001
Temporal (2.0)	192.4 ± 51.3	261.1 ± 30.4	<0.001
Temporal (1.5)	193.1 ± 50.1	255.4 ± 35.3	<0.001
Temporal (1.0)	186.5 ± 53.8	245.3 ± 37.2	<0.001
Temporal (0.5)	178.4 ± 53.0	231.2 ± 44.0	<0.001
Fovea (0)	167.3 ± 53.6	223.2 ± 39.3	<0.001
Nasal (0.5)	153.3 ± 50.0	210.4 ± 40.3	<0.001
Nasal (1.0)	139.2 ± 47.9	192.7 ± 43.0	<0.001
Nasal (1.5)	119.2 ± 43.9	174.1 ± 40.4	<0.001
Nasal (2.0)	99.8 ± 40.2	151.0 ± 36.0	<0.001
Nasal (2.5)	82.4 ± 35.8	125.7 ± 32.3	<0.001

**Table 3:** Mean choroidal thickness in the vertical direction.

Location (mm from fovea)	Tessellated fundus eyes	Normal fundus eyes	*P* value
	Mean CT ± SD, *μ*m	Mean CT ± SD, *μ*m	
Inferior (2.5)	180.3 ± 54.1	221.6 ± 37.7	0.001
Inferior (2.0)	180.8 ± 51.6	226.1 ± 40.2	<0.001
Inferior (1.5)	176.1 ± 50.1	225.9 ± 38.2	<0.001
Inferior (1.0)	170.9 ± 50.5	225.4 ± 36.6	<0.001
Inferior (0.5)	169.7 ± 51.0	231.7 ± 36.0	<0.001
Fovea (0)	164.5 ± 52.0	224.1 ± 39.8	<0.001
Superior (0.5)	175.2 ± 51.8	232.2 ± 41.5	<0.001
Superior (1.0)	182.4 ± 54.8	238.0 ± 40.3	<0.001
Superior (1.5)	196.9 ± 55.9	257.8 ± 32.9	<0.001
Superior (2.0)	206.2 ± 54.5	256.9 ± 43.1	<0.001
Superior (2.5)	213.7 ± 56.5	257.9 ± 43.7	0.001

**Table 4:** Stepwise multiple linear regression model of subfoveal choroidal thickness.

Factors	Beta (95%, confidence interval)	*P* value
Normal fundus (versus tessellation fundus)	54.61 (29.55, 79.67)	<0.001
Axial length (mm)	−14.31 (−27.06, −1.56)	0.028
Age (year)	−1.60 (−2.50, −0.71)	0.001
Gender (male versus female)	29.39 (8.98, 49.80)	0.005

**Table 5:** Stepwise multiple linear regression analysis of the choroidal thickness in various loci (adjusted for axial length, age, and gender).

Loci	Mean difference between C_1_ and C_0_ groups (*μ*m)	95% confidence interval		*P* value
		Lower limit	Upper limit	
SFCT	−54.61	−79.67	−29.55	<0.001
Temporal (2.5)	−68.71	−91.21	−46.20	<0.001
Temporal (2.0)	−68.70	−91.31	−46.10	<0.001
Temporal (1.5)	−62.30	−84.92	−39.68	<0.001
Temporal (1.0)	−58.80	−82.79	−34.81	<0.001
Temporal (0.5)	−52.82	−77.00	−28.65	<0.001
Nasal (0.5)	−57.13	−79.85	−34.42	<0.001
Nasal (1.0)	−53.50	−75.60	−31.40	<0.001
Nasal (1.5)	−54.87	−74.74	−35.00	<0.001
Nasal (2.0)	−51.27	−69.81	−32.728	<0.001
Nasal (2.5)	−43.29	−59.83	−26.74	<0.001
Inferior (2.5)	−41.33	−65.51	−17.14	<0.001
Inferior (2.0)	−45.35	−68.70	−22.00	<0.001
Inferior (1.5)	−49.80	−72.55	−27.05	<0.001
Inferior (1.0)	−54.52	−76.75	−32.30	<0.001
Inferior (0.5)	−61.96	−84.76	−39.17	<0.001
Superior (0.5)	−57.01	−80.51	−33.50	<0.001
Superior (1.0)	−55.64	−80.24	−31.04	<0.001
Superior (1.5)	−60.85	−85.46	−36.24	<0.001
Superior (2.0)	−50.72	−75.40	−26.04	<0.001
Superior (2.5)	−44.20	−69.74	−18.66	0.001

SFCT: subfoveal choroidal thickness; C_0_: normal fundus; C_1_: tessellated fundus.

**Table 6:** Risk factors associated with highly myopic tessellation.

Factors	Unadjusted OR (95%, CI)	*P* value	Adjusted OR (95%, CI)	*P* value
Axial length	7.597 (2.547, 22.549)	<0.001	5.622 (1.769, 17.872)	0.003
SFCT	0.979 (0.966, 0.991)	0.001	0.975 (0.960, 0.990)	0.001

SFCT: subfoveal choroidal thickness; OR: odds ratio; CI: confidence interval.

**Table 7:** Basic biometrics of age- and gender-matched subgroups.

	Tessellated fundus eyes	Normal fundus eyes	*P* value
No. of eyes	44	22	—
Mean age, year	37.3 ± 10.5	37.2 ± 9.9	0.953
IOP at imaging, mmHg	15.76 ± 2.40	16.01 ± 2.59	0.705
Axial length, mm	27.11 ± 0.78	26.12 ± 0.74	<0.001^∗^
Spherical equivalent, diopter	−9.242 ± 1.585	−7.882 ± 1.017	0.001^∗^
Subfoveal choroidal thickness, *μ*m	150.96 ± 49.9	223.6 ± 39.3	<0.001^∗^
Macular choroidal thickness, *μ*m	158.92 ± 44.1	224.3 ± 29.1	<0.001^∗^
Foveal retinal thickness, *μ*m	190.7 ± 14.6	192.9 ± 14.7	0.849
Central subfield thickness, *μ*m	246.4 ± 18.4	245.0 ± 21.0	0.787

Data are demonstrated as mean ± SD. IOP: intraocular pressure. ^∗^*P* value < 0.05.

**Table 8:** AUC, sensitivity, and specificity of various biometric parameters in detecting highly myopic tessellation.

Factor	AUC (95%, CI)	*P* value	Sensitivity (%)	Specificity (%)
SFCT	0.824 (0.747, 0.901)	<0.001	81.8	74.2
AL	0.820 (0.716, 0.924)	<0.001	78.5	77.3
RE	0.639 (0.529, 0.749)	0.043	81.8	50.5
FRT	0.528 (0.390, 0.666)	0.688	77.4	36.4

AUC: area under the curve; CI: confidence interval; SFCT: subfoveal choroidal thickness; AL: axial length; RE: refractive error; FRT: foveal retinal thickness.
